# Effect of individual and community-level bed net usage on malaria prevalence among under-fives in the Democratic Republic of Congo

**DOI:** 10.1186/s12936-018-2183-y

**Published:** 2018-01-18

**Authors:** Lauren Levitz, Mark Janko, Kashamuka Mwandagalirwa, Kyaw L. Thwai, Joris L. Likwela, Antoinette K. Tshefu, Michael Emch, Steven R. Meshnick

**Affiliations:** 1 Department of Epidemiology, University of North Carolina, Gillings School of Global Public Health, 135 Dauer Drive, 3113 Michael Hooker Research Building, Chapel Hill, NC 27599 USA; 2 Department of Geography, University of North Carolina Gillings School of Global Public Health, Chapel Hill, NC 27599 USA; 3 University of Kinshasa School of Public Health, Kinshasa, Democratic Republic of Congo; 4 Programme National de la Lutte contre le Paludisme, Kinshasa, Democratic Republic of Congo

**Keywords:** Malaria, Mosquito nets, ITNs, LLINs, DRC, Democratic Republic of Congo, Under-fives, Insecticide resistance

## Abstract

**Background:**

Understanding the contribution of community-level long-lasting, insecticidal net (LLIN) coverage to malaria control is critical to planning and assessing intervention campaigns. The Democratic Republic of Congo (DRC), which has one of the highest burdens of malaria cases and deaths and has dramatically scaled up LLIN ownership in recent years thus it is an ideal setting to evaluate the effect of individual versus community-level use to prevent malaria among children under the age of 5.

**Results:**

Data were derived from the 2013–2014 DRC Demographic and Health Survey. Community-level LLIN usage was significantly associated with protection against malaria, even when individual-level LLIN usage was included in the model. In stratified analysis, higher levels of community LLIN coverage enhanced the protective effect of individual LLIN usage, resulting in lower malaria prevalence among individuals who used a LLIN. A sub-analysis of individual LLIN usage by insecticide type revealed deltamethrin-treated nets were more protective than permethrin-treated nets, suggesting that mosquitoes in the DRC are more susceptible to deltamethrin.

**Conclusions:**

This study examines the effects of individual and community-level LLIN usage in young children in an area of high ITN usage. Individual and community LLIN usage were significantly associated with protection against malaria in children under 5 in the DRC. Importantly, the protective effect of individual LLIN usage against malaria is enhanced when community LLIN coverage is higher, demonstrating the importance of increasing community-level LLIN usage. LLINs treated with deltamethrin were shown to be more protective against malaria than LLINs treated with permethrin. Demographic and Health Surveys are thus a novel and important means of surveillance for insecticide resistance.

## Background

Malaria causes an estimated 214 million cases and 438,000 deaths yearly, of which the majority occur in children < 5 years old living in Africa [1, 2]. Insecticide-treated bed nets (ITNs) are a key vector control intervention. ITNs work in three ways: by blocking exposure to potentially infective mosquito bites (preventing transmission from mosquito to human), by preventing contact between a mosquito and a malaria-infected individual (preventing transmission from human to mosquito), and by killing mosquitoes that come into contact with them [3]. The World Health Organization (WHO) estimates ITNs are responsible for preventing 69% of the 663 million malaria cases averted due to malaria control interventions between 2001 and 2015 [1]. Since 2007, the WHO has recommended that all ITNs be long-lasting insecticidal nets (LLINs), which are constructed to retain insecticidal activity for at least 20 standard washes under laboratory conditions and 3 years of use in the field [4]. LLINs have been shown to significantly reduce the odds of infection and prevent clinical malaria in children in various settings [5, 6].

While LLINs are an intervention targeted to individuals, coverage of a certain percentage of the population might provide a community effect. Previous studies examining ITN community coverage have shown an association with decreased risk of malaria [7–10]. However, such studies tend to be small, lack generalizability, and often focus on adults or older children. Much of the literature comes from clinical trials or from modelling rather than from research done in the field [11–13]. Furthermore, little research has been done in areas of high ITN usage. Understanding the contribution of community-level ITN coverage to malaria risk is critical to planning and assessing intervention campaigns and national control strategies.

The Democratic Republic of Congo (DRC) has one of the highest burdens of malaria cases and deaths, second only to Nigeria [1]. Lack of access to health care, a majority rural population, high poverty, and political instability are contributors to the malaria endemicity [14]. The DRC began distributing ITNs in 2007 [15], and reported ITN ownership increased dramatically by 2013: the percentage of households reporting ownership of at least one net increased from 28 to 72% for ITNs and from 9 to 70% for LLINs [16]. By 2014, over a third of all malaria intervention funds (government, Global Fund, President’s Malaria Initiative, WHO, United Nations International Children’s Emergency Fund, others) went toward ITNs [1]. Given the high malaria burden and current interventional focus on ITNs, the DRC is an ideal setting in which to evaluate the effect of individual versus community-level LLIN usage in preventing malaria.

## Methods

Data were obtained from the second DRC Demographic and Health Survey (DHS) conducted from November 2013 to February 2014 [16]. A total of 536 clusters across all 26 health areas (formerly 11 provinces) were surveyed, comprising 18,360 households. Clustered sampling was designed to allow indicators to be representative at the national level, provincial level, and for urban and rural areas. For 492 of the clusters, global positioning system (GPS) coordinates were recorded using a random displacement method to prevent participant identification. The DHS was unable to collect GPS coordinates from the remaining 44 clusters. Rapid diagnostic tests (RDTs) for malaria and malaria-specific questionnaires were administered to all children ages 6–59 months in half of the study-eligible households.

**Fig. 1:**
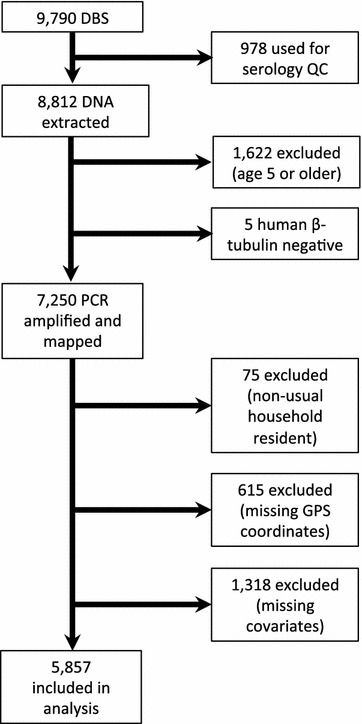
Enrollment criteria for study inclusion. A total of 9790 dried blood spots (DBS) were collected from children included in the second Demographic And Health Survey in the Democratic Republic of Congo (DHS-DRC II) from November 2013 to February 2014. A complete case analysis was performed on the 5857 samples from children ages 59 months or younger

Dried blood spots (DBS) were collected, stored, and shipped to the University of North Carolina for polymerase-chain reaction (PCR) analysis. DNA was extracted from DBS from 9790 children using Chelex [17], and PCR amplification for *Plasmodium falciparum* lactate dehydrogenase (DNA) was conducted as described previously [18], with human β-tubulin as a DNA control [19]. Children who were older than 59 months, were missing GPS coordinates or malaria PCR results, or who were reported to the DHS-DRC II to not be usual residents of the surveyed household, were excluded from further analysis. Since a complete case analysis was performed, children who were missing covariate data were also excluded (Fig. 1).

Data were available for a total of 5857 children; they were included in assessment of multivariate models. Individual- and community-level risk factors were identified a priori based on a review of the literature. Age was defined as the reported age of the child in months. The wealth index was defined by the DHS as economic wellbeing quintiles (lowest, second, middle, fourth, highest), based on self-reported ownership of certain goods (television, radio, car, etc.) and certain household characteristics (including electricity, type of drinking water supply, toilet type, number of sleeping rooms, type of cooking fuel) [16]. Poor household construction is a known risk factor for malaria acquisition [20]; thus a household index was constructed based on survey responses. The household index was coded from 0 to 6, with two points assigned for “finished,” one for “rudimentary,” and zero for “natural” for each of the three housing categories (roof, floor, walls). Mother’s highest education level was coded from 0 to 3, corresponding to no education, primary school, secondary school, or higher education. Regional-level 2007 malaria prevalence was calculated by plotting the cluster GPS coordinates onto the 2007 *P. falciparum* malaria prevalence map previously [21]. Malaria prevalence was dichotomized as low (< 50%) or high (≥ 50%). This variable was assessed for inclusion because malaria is persistent in space over time, and historical malaria prevalence might influence current LLIN usage. Community LLIN usage was calculated as the percentage of children within a given cluster who reported using a LLIN the previous night divided by the total number of observations in that cluster. Individual LLIN usage the previous night was coded as a dichotomous yes/no for LLIN usage the previous night. Children who reported using an untreated net were coded as not having used a LLIN the night before.

In order to assess both individual and community level variables, multilevel models were constructed. Multilevel models adjust for the collinearity of individual and cluster-level variables. Three crude multilevel logistic regression models were constructed to test association with malaria infection: one with individual LLIN usage (Model 1), one with community LLIN usage (Model 2), and one with both (full model). The outcome variable was a dichotomous positive/negative malaria diagnosis for *P. falciparum* as assessed by real-time PCR. The models were built in SAS v. 9.4 (SAS Institute, Cary, NC) using PROC SURVEYLOGISTIC adjusted for DHS-DRC II sampling weights. A multilevel modelling approach is necessary to take into account the correlation of individuals within clusters. Expected relationships between risk factors and malaria diagnosis were defined a priori. Non-significant variables were removed from the model by backwards selection. The three models were assessed by Akaike’s Information Criterion (AIC), which penalizes over-fitting and favours parsimony, with lower AIC values preferred. Model 1 included the following variables: age in months, housing index, mother’s highest educational level, individual LLIN usage, and cluster altitude in meters. Model 2 included the following variables: age in months, housing index, mother’s highest educational level, cluster LLIN usage, and cluster altitude in meters. Model 3 included the following variables: age in months, housing index, mother’s highest educational level, individual LLIN usage, cluster LLIN usage, and cluster altitude in meters.

It is plausible that individuals who did not sleep under a LLIN might benefit if there is a protective effect of community LLIN usage. While previous studies have demonstrated a positive association between community LLIN usage ≥ 50% and decreased malaria infection among children in households possessing at least one ITN [22, 23], there is not a definitive threshold that is protective. Thus to determine whether the effect of individual LLIN usage is modified by varying levels of community-level LLIN usage, the community-level LLIN usage variable was stratified into quartiles. Three interaction terms between the community-level coverage variables and individual-level LLIN usage was included to test for the presence of effect modification of the odds ratio. Homogeneity of the stratum-specific exposure odds ratio estimates was assessed by likelihood ratio test (LRT) at an a priori level of 0.05.

For those who reported using a LLIN the previous night, LLIN brand was also reported. LLIN brands were recoded by insecticide type as follows: deltamethrin (PermaNet, Yorkool LN, Lifenet, Serena, Netprotect); permethrin (Olyset); alpha-cypermethrin (Duranet, Magnet, Interceptor); and brand not specified. Model 1 was modified to test the association between individual use of a LLIN treated with a particular insecticide (deltamethrin, permethrin, alpha-cypermethrin, or brand not specified) and odds of PCR-positive malaria; the four insecticides were tested in four separate models, with no LLIN usage as the referent group.

## Results

Of the 5857 individuals included in this study, 37.4% were PCR-positive for *P. falciparum* and 53.6% had slept under a bed night the previous night. A summary of the prevalences of malaria by covariate is presented in Table 1. Factors which were significantly associated with malaria prevalence include age, housing quality, wealth, mother’s education, individual and community of bednets, prevalence in 2007, rurality, type of bed net insecticide, and altitude. No significant differences were seen by gender or bednet age.

**Table 1:** Prevalence of PCR-positive malaria among subjects

Variable (n)	% PCR positive	*p* value*
Age		
< 1 year (658)	29	< 0.001
1–2 years (1388)	31	
2–3 years (1303)	38	
3–4 years (1312)	42	
4–5 years (1189)	43	
Sex		
Male (2935)	38	0.365
Female (2915)	37	
Housing quality index		
Lowest quality (786)	31	< 0.001
2nd level (3118)	42	
3rd level (365)	40	
4th level (403)	35	
5th level (459)	34	
6th level (71)	25	
Highest quality (648)	23	
Wealth index		
Lowest quintile (1558)	40	< 0.001
2nd quintile (1366)	40	
Middle quintile (1153)	39	
4th quintile (1034)	39	
Highest quintile (739)	22	
Mother’s education		
None (1255)	41	< 0.001
Primary (2633)	41	
Secondary (1903)	30	
Higher (59)	19	
Number of household members		
2–4 (1191)	36	0.505
5–7 (2629)	37	
8–10 (1510)	39	
> 10 (520)	36	
Respondent slept under LLIN previous night		
Yes (2700)	42	< 0.001
No (3150)	34	
Altitude (m)		
< 500 (2165)	38	< 0.001
500–1000 (2609)	42	
1000–1500 (629)	35	
1500–2000 (447)	9	
2007 prevalence		
High (2783)	44	< 0.001
Low (3067)	32	
% Cluster LLIN coverage previous night (quartiles)		
0–33 (1202)	42	< 0.001
34–54 (1688)	40	
55–75 (1495)	36	
76–100 (1321)	31	
Time to water source (min)		
0–12 (1222)	37	0.089
12–30 (2304)	40	
30–45 (662)	35	
Over 45 (1400)	37	
Urban/rural residence		
Urban (1772)	35	0.012
Rural (4078)	38	
Bed net age		
< 1 year (824)	34	0.055
1–2 years (1440)	31	
2–3 years (479)	36	
> 3 years (475)	38	
Insecticide		
Alphacypermethrin (59)	24	< 0.001
Deltamethrin (2592)	32	
Permethrin (445)	42	

* Chi square test for null hypothesis that all prevalences are equal

Six multilevel models were considered: three crude models (full model including both individual- and community-level LLIN usage, Model 1 with only individual-level LLIN usage, and Model 2 with only community-level LLIN usage) and three adjusted models. Only variables that were significant at an alpha level of p < 0.05 were included in a given adjusted model. Covariates that were significant in all three models include age in months, housing index, mother’s highest educational level, and cluster altitude.

Prevalence odds ratios (OR) for the three models are presented in Table 2. The crude models include only individual-level LLIN usage, community-level LLIN usage, or both. The adjusted models include covariates identified as significant as described in Methods. In both Model 1 and Model 3, individual LLIN usage resulted in a significant decrease in the OR of PCR-positive malaria. This held true after adjustment for covariates. In Models 2 and 3, community-level LLIN usage significantly reduced the odds of PCR-positive malaria after adjustment for covariates, but not in the crude models. The best fitting model as determined by the lowest AIC was Model 3 adjusted for covariates (AIC 7127.47), which includes both individual—(OR 0.77, 95% CI 0.66, 0.91) and community-level LLIN usage (OR 0.56, 95% CI 0.33, 0.95).

**Table 2:** Comparison of odds ratios and model fit across the three models

	Model 1: individual LLIN use		Model 2: community LLIN use		Model 3: both	
	Crude	Adjusted	Crude	Adjusted	Crude	Adjusted
Odds ratio (95% CI)						
Individual LLIN use	0.72 (0.61, 0.87)	0.65 (0.55, 0.77)			0.75 (0.64, 0.88)	0.77 (0.66, 0.91)
Community LLIN use			0.67 (0.40, 1.12)	0.43 (0.27, 0.70)	0.89 (0.51, 1.55)	0.56 (0.33, 0.95)
*AIC*	7438.04	7127.89	7455.49	7121.07	7439.11	7109.15

To determine whether the effect of individual LLIN usage is modified by varying levels of community-level LLIN usage, the community-level coverage variable was stratified into quartiles. This was coded as three disjoint indicator variables and added to the final model (Model 3, adjusted). Stratum-specific odds ratio estimates are presented in Table 3. There is a trend toward an increased protective effect of individual LLIN usage as community-level LLIN usage increases. Individual LLIN usage is most protective against PCR-positive malaria at the highest quartile of community-level LLIN usage (OR 0.47, 95% CI 0.30, 0.73). The test for homogeneity was significant, indicating there is a departure from perfect multiplicativity of the odds ratio, but there is a high degree of overlap of the 95% confidence intervals for the stratum-specific estimates. Notably, higher community-level LLIN usage led to a decrease in malaria prevalence, but only among those who used a LLIN themselves, indicating the importance of individual LLIN usage.

**Table 3:** Effect of community LLIN use on the protective effect of individual LLIN use

Quartile of community-level LLIN usage	Individual LLIN OR (95% CI)	Malaria prevalence (%)		LRT statistic	DF	*p* value
		Individual LLIN	No individual LLIN			
Lowest quartile	0.81 (0.58, 1.15)	39.4	42.4	12.37	3	0.01
Second quartile	0.87 (0.68, 1.11)	38.5	42.7			
Third quartile	0.71 (0.53, 0.95)	34.1	39.4			
Highest quartile	0.47 (0.31, 0.69)	29.2	42.3			

**Fig. 2:**
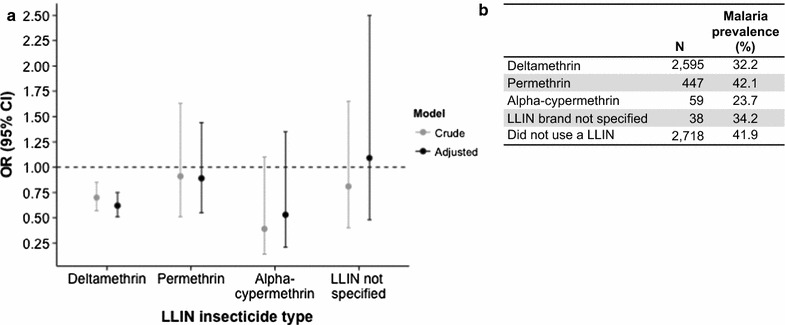
Effect of LLIN insecticide type on PCR-positive malaria. LLINs were categorized by type of insecticide based on brand name as follows: deltamethrin (PermaNet, Yorkool LN, Lifenet, Serena, Netprotect); permethrin (Olyset); alpha-cypermethrin (Duranet, Magnet, Interceptor); or LLIN brand not specified. **a** Crude and adjusted (for age in months, housing index, maternal education, and altitude) odds ratios are presented by individual use of each LLIN type (versus no LLIN). Only deltamethrin-treated LLINs significantly reduced the odds of PCR-positive malaria in both the crude and adjusted models. **b** Number of subjects using each type of net and prevalence of malaria among them

Prevalence odds ratio estimates for individual LLIN usage by insecticide type are shown in Fig. 2a (with raw data in Fig. 2b). Use of a deltamethrin-treated net significantly reduced the odds of PCR-positive malaria as compared to no LLIN use in both crude (OR 0.70, 95% CI 0.57, 0.85) and adjusted (OR 0.62, 95% CI 0.51, 0.75) models. Alpha-cypermethrin-treated nets led to even lower ORs, but these were not significant in the crude (OR 0.39, 95% CI 0.14, 1.10) or adjusted (OR 0.53, 95% CI 0.21, 1.35) models, likely due to very small sample size. Permethrin-treated nets led to non-significant ORs close to the null in both crude (OR 0.91, 95% CI 0.51, 1.63) and adjusted (OR 0.89, 95% CI 0.55, 1.44) models. Malaria prevalence among those who used deltamethrin-treated nets was 32.2%, as compared to 42.1% among those who used permethrin-treated nets and 41.9% among those who did not use a LLIN (Fig. 2b). The observed differences were not due to bed net age; the median reported ages (IQR) of the bednets were 13 (11–24) months, 16 (12–24) months and 13 (12–24) months, for deltamethrin-, alpha-cypermethrin- and permethrin-treated nets, respectively, and were not significantly different.

## Discussion

These results demonstrate the importance of both individual- and community-level LLIN usage in preventing malaria among those most at risk of infection in sub-Saharan African, children under the age of 5. Furthermore, this study examines the effects of individual as well as community-level LLIN usage in young children in an area of high ITN usage. High LLIN usage was significantly associated with protection against malaria, even when individual-level LLIN usage was included in the model. Importantly, higher levels of community LLIN coverage enhanced the protective effect of individual LLIN usage, resulting in lower malaria prevalence among individuals who used a LLIN. Deltamethrin-treated nets were more protective than permethrin-treated nets. However, since the nets were not randomly distributed, the observed differences could have been affected by selection bias.

The significant effect of individual-level LLIN usage on odds of malaria is different from what was observed in the DRC in 2007, where ITN usage was found to be protective at the community level but not at the individual level [9]. This could be due to several reasons. First, the 2007 DHS-DRC survey included adults ages 15–59 and excluded children [9] whereas the current study population is exclusively children. Adults are more likely to be outside during peak-biting evening hours [24–26], potentially increasing their exposure to malarial mosquitoes. Consequently, community-level ITN coverage might be more important for malaria prevention at older ages. Second, the distribution of treated versus untreated nets in the DRC has shifted substantially since the 2007 DHS-DRC: while roughly twice as many survey respondents reported sleeping under an untreated net as compared to a treated net in the 2007 DHS-DRC [9], the vast majority of DHS-DRC II respondents slept under a treated net [16]. This dramatic increase in ITNs and LLINs from 2007 to 2013–2014 could lead to a larger individual protective effect and reduce the relative importance of community coverage. The effect of individual LLIN usage is modified by community-level LLIN usage, and stratified analysis indicates that individual net usage is more protective when community coverage is high.

Despite mass distribution campaigns, LLIN usage remains below full coverage of the at-risk population. Factors such as household wealth, age, educational status of the head of the household, number of children under-five, malaria endemicity, and distance to health centers have been demonstrated to affect children’s ITN usage in other African settings [27]. Education campaigns have been shown to significantly increase ITN usage [28, 29], and cost of ITN (free versus subsidized versus full market price) influences ITN ownership though likely not ITN usage [29].

The 2013–2014 DHS-DRC did not obtain information about why survey respondents did or did not choose to use a LLIN. Based on results from the full 2013–14 DHS-DRC, overall access to an ITN in the DRC was found to be 47%, with access dependent on several factors, including province (ranging from 62.6% in Bandundu to 31% in Kasai-Occidental) and number of people sleeping in the household (ranging from 63.7% for 2 people to 38.1% for 8 people) [16]. In order to make policy recommendations about future ITN campaigns, more research is needed to determine geographic areas to prioritize and methods to improve ITN usage in this population following distributions. This would allow for the development of highly targeted ITN campaigns paired with culturally appropriate education campaigns.

Insecticide resistance is a growing concern, and pyrethroid resistance among *Anopheles* mosquitoes has been reported in the DRC [30–32]. The three most commonly used LLIN brands reported in the 2013–14 DHS-DRC are treated with deltamethrin (PermaNet, Serena) or permethrin (Olyset). A field efficacy trial in Kinshasa demonstrated significantly higher bioefficacy of PermaNet 3.0 LLINs against *Anopheles gambiae* mosquitoes from the DRC as compared to OlysetNet; furthermore, individuals sleeping under PermaNet 3.0 LLINs had lower biting rates and reported better sleep quality as compared to those sleeping under OlysetNet [32]. The authors note, however, that the improved effectiveness of PermaNet 3.0 might be attributed to both the deltamethrin and the addition of piperonyl butoxide (PBO), which increases insecticide penetration and inhibits metabolic detoxification [32]. Given these results, it is encouraging that the vast majority of LLINs used by children in the current study were PermaNet or other deltamethrin-treated brands. As examination of insecticide resistance or bioefficacy of LLINs was outside the scope of the current project, the observed increased protection of deltamethrin-treated nets might also be attributed to additional factors such as age and condition of the net or distribution of permethrin-treated nets to higher-transmission areas. Phenotypic resistance to all three pyrethroids has been documented in field-collected *A. gambiae* mosquitoes from the DRC [31]. If there is a high degree of insecticide resistance in the study area, the effect of community-level LLIN usage on the odds of PCR-positive malaria might be diminished due to reduced killing of infectious mosquitoes that come into contact with treated nets. Consequently, individual use of a bed net might become more important, as the nets would act as a barrier to prevent exposure even if the insecticides are less effective. This is supported by the increased effect size of individual LLIN usage on the odds of PCR-positive malaria among communities with higher as compared to lower levels of LLIN coverage. Examination of insecticide resistance is an important area for future research.

This study was limited by the cross-sectional design of the DHS-DRC. Cross-sectional studies are useful in identifying associations but are unable to establish causality. Malaria prevalence (and likely ITN usage) varies seasonally, meaning that the effect of individual versus community-level ITN usage on malaria risk might depend on season as well. A longitudinal study in the Kinshasa province is currently underway, which will be able to address such questions.

## Conclusions

Individual and community usage of LLINs is significantly associated with protection against malaria in children under the age of 5. Importantly, the protective effect of individual LLIN usage against malaria is enhanced when community LLIN coverage is higher, demonstrating the importance of increasing community-level LLIN usage. LLINs treated with deltamethrin were shown to be more protective against malaria than LLINs treated with permethrin. Thus, DHS surveys are a rich potential sources of information about insecticide resistance.

## Abbreviations

DBS: dried blood spot
DHS: Demographic and Health Survey
DRC: Democratic Republic of the Congo
ITN: insecticide-treated net
LLIN: long-lasting, insecticidal net
PCR: polymerase-chain reaction
RDT: rapid diagnostic test
WHO: World Health Organization
